# Functional Connectivity Changes After Initial Treatment With Fingolimod in Multiple Sclerosis

**DOI:** 10.3389/fneur.2019.00153

**Published:** 2019-03-22

**Authors:** Nikolaos Petsas, Laura De Giglio, Vicente González-Quintanilla, Manuela Giuliani, Floriana De Angelis, Francesca Tona, Maurizio Carmellini, Caterina Mainero, Carlo Pozzilli, Patrizia Pantano

**Affiliations:** ^1^Department of Radiology, IRCCS NEUROMED, Pozzilli, Italy; ^2^Multiple Sclerosis Centre, Azienda Ospedaliera Sant'Andrea, Rome, Italy; ^3^Department of Human Neurosciences, Sapienza University of Rome, Rome, Italy; ^4^Department of Neurology, University Hospital Marquis of Valdecilla, Santander, Spain; ^5^Queen Square Multiple Sclerosis Centre, UCL Queen Square Institute of Neurology, University College London, London, United Kingdom; ^6^Department of Radiology, Athinoula A. Martinos Center for Biomedical Imaging, Massachusetts General Hospital, Charlestown, MA, United States; ^7^Harvard Medical School, Boston, MA, United States

**Keywords:** multiple scleorsis (MS), resting-state functional MRI, functional connectivity, fingolimod (FTY720), motor task

## Abstract

On the basis of recent functional MRI studies, Multiple Sclerosis (MS) has been interpreted as a multisystem disconnection syndrome. Compared to normal subjects, MS patients show alterations in functional connectivity (FC). However, the mechanisms underlying these alterations are still debated. The aim of the study is to investigate resting state (RS) FC changes after initial treatment with fingolimod, a proven anti-inflammatory and immunomodulating agent for MS. We studied 32 right-handed relapsing-remitting MS patients (median Expanded Disability Status Scale: 2.0, mean disease duration: 8.8 years) who underwent both functional and conventional MRI with a 3 Tesla magnet. All assessments were performed 3 weeks before starting fingolimod, then, at therapy-initiation stage and at month 6. Each imaging session included scans at baseline (run1) and after (run2) a 25-min, within-session, motor-practice task, consisting of a paced right-thumb flexion. FC was assessed using a seed on the left primary motor cortex to obtain parametric maps at run1 and task-induced FC change (run2-run1). Comparison between 3-week before- and fingolimod start sessions accounted for a test-retest effect. The main outcome was the changes in both baseline and task-induced changes in FC, between initiation and 6 months. MRI contrast enhancement was detected in 14 patients at initiation and only in 3 at month 6. There was a significant improvement (*p* < 0.05) in cognitive function, as measured by the Paced Auditory Serial Addition Task, at month 6 compared to initiation. After accounting for test-retest effect, baseline FC significantly decreased at month 6, with respect to initiation (*p* < 0.05, family-wise error corrected) in bilateral occipito-parietal areas and cerebellum. A task-induced change in FC at month 6 showed a significant increment in all examined sessions, involving not only areas of the sensorimotor network, but also posterior cortical areas (cuneus and precuneus) and areas of the prefrontal and temporal cortices (*p* < 0.05, family-wise error corrected). Cognitive improvement at month 6 was significantly (*p* < 0.05) related to baseline FC reduction in posterior cortical areas. This study shows significant changes in functional connectivity, both at baseline and after the execution of a simple motor task following 6 months of fingolimod therapy.

## Introduction

Multiple Sclerosis (MS) is a chronic neurologic disease and a leading cause of disability in young adults (1). Independently from the cause of MS, disability and disease burden in the single patient may not coincide, suggesting that mechanisms of repair and compensation could play a relevant role on clinical outcome (2). In recent years, functional MRI (fMRI) has contributed to the understanding of the complex mechanisms of the disease in relation to brain tissue damage and the different stages of the disease (3).

FMRI, performed in a resting-state (RS) condition, allows the investigating of the brain spontaneous neuronal activity (4), avoiding the variable effect of disability in task-fMRI performance. This spontaneous brain activity manifests as slow fMRI signal fluctuations that are synchronized across anatomically separate, but functionally connected, brain regions, a coherence that represents the brain's RS functional connectivity (FC) (4, 5). RS activity can be modified by internal and external stimuli and can be conditioned by any task (6).

Published studies generally agree that FC in MS differs significantly with respect to healthy controls in most large-scale networks (7, 8), but the pathophysiological and, consequently, the clinical meaning of these observations is uncertain (9). In general, higher FC has been interpreted either as an immediate expression of reaction to damage or a longer maladaptive response of reduced network efficiency (9) and it is often correlated with worse performance, particularly in cognitive functions (10, 11).

By applying a motor task between two consecutive RS-fMRI sessions, we have recently disclosed, in early MS cases compared with healthy subjects, significant changes in FC in motor-related networks that were not otherwise present in the baseline RS condition, before the motor training task (12). Those changes were induced or conditioned by the motor task in the sensorimotor and cerebellar networks and represent an aspect of functional modulation or reactivity, as a response at the level of large-scale RS networks.

We have also found that MS inflammation alters short-term plasticity underlying a motor-practice task, consisting in repetitive right thumb-flexions, between two fMRI sessions, and that the pharmacological modulation of inflammation in MS with Interferon treatment may promote brain functional reorganization during a motor task (13).

Fingolimod (FTY–Gilenya; Novartis Pharma AG, Basel, Switzerland), an oral therapy available for patients with relapsing-remitting (RR) MS (14), has demonstrated superior efficacy with respect to relapse rates and MRI outcomes over intramuscular interferon beta-1a (15). Experimental evidence suggests that the anti-inflammatory and immuno-modulatory effects of fingolimod may result in the restoration of synaptic plasticity and the facilitation of recovery (16, 17).

Here, in patients suffering from RRMS, we investigated whether FC, both at baseline and after the performance of a simple motor task, may be modified by fingolimod therapy.

## Materials and Methods

### Study Design

**Figure 1:**
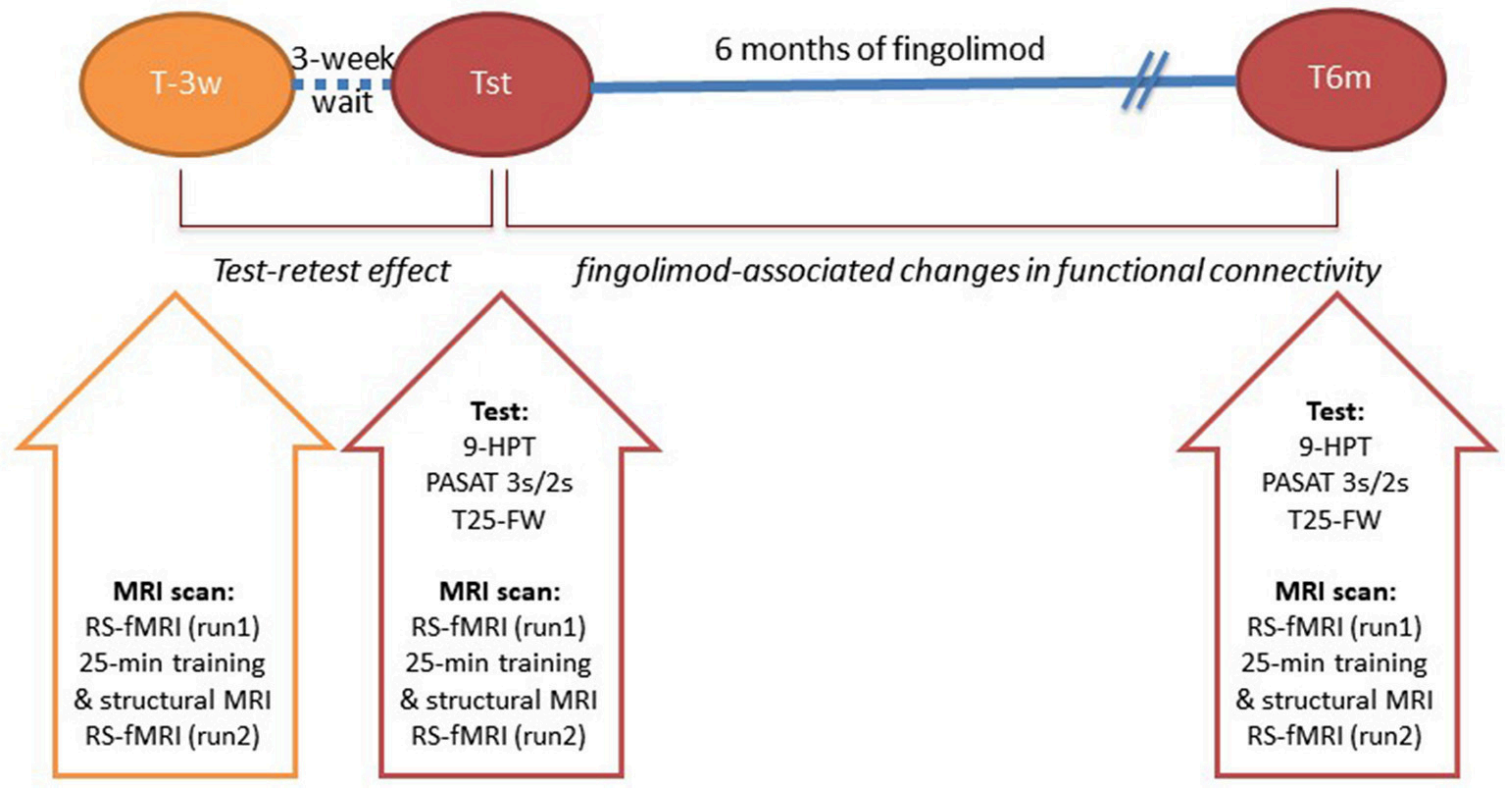
Study Design: patients were assessed in three different sessions with respect to the fingolimod treatment: 3 weeks before initiation (T-3w), a day or two before fingolimod therapy initiation (Tst) and at 6 months of fingolimod treatment (T6m). Abbreviations: 9-Hole Peg Test (9HPT); Paced Auditory Serial Addition Test (PASAT) at 3 and seconds; 25-Feet Walk Test (25-FWT); resting-state functional MRI (RS-fMRI).

This was a prospective interventional study. Patients were assessed using clinical and MRI measures at three different time points (see Figure 1): (1) 3 weeks before starting fingolimod treatment (T-3w); (2) 1–2 days prior to starting fingolimod (Tst); (3) after 6 months of fingolimod treatment (T6m).

#### Participants

A consecutive series of patients with a diagnosis of RRMS (18) were recruited at the Sant'Andrea Hospital, Sapienza University of Rome, Italy and underwent an MRI scan and clinical testing at the Policlinico Umberto I, Sapienza University of Rome, Italy.

Inclusion criteria were: eligibility to be treated with fingolimod according to the European Medicines Agency indication; age 18–50 years; right-handedness.

Exclusion criteria were: supraventricular or ventricular arrhythmia detected at 12-lead ECG; a history of previous cardiovascular disease, including hypertension, coronary artery disease, congestive heart failure, severe valvular heart disease; hyperthyroidism; electrolyte imbalance; chronic kidney dysfunction (estimated glomerular filtration rate <60 ml/min by the Cockroft-Gault formula); concomitant treatment with cardiovascular and/or non-cardiovascular drug that may alter sinus rhythm and heart rate; other neurological or psychiatric disease.

At each time point, before being scanned, participants were assessed by a trained physician and the following clinical outcome were collected: Expanded Disability Status Scale (EDSS) (19), MS Functional Composite (MSFC) score with its sub-scores 9-Hole Peg Test (9HPT), 25-Feet Walk Test (25-FWT), and the Paced Auditory Serial Addition Test (PASAT) 3 and 2 s. The following patient-reported outcome measures were also collected: Multiple Sclerosis Quality of Life—54 items (MSQoL-54) Italian versions, Multiple Sclerosis Impact Scale-29 items (MSIS-29) and Beck's Depression Inventory-II.

### MRI Acquisition

Images were acquired with a Siemens Magnetom Verio 3 Tesla scanner and a 12-channel head coil designed for parallel imaging (GRAPPA). Participants were advised to avoid consuming psychoactive substances, such as tea or coffee, in the 2 h before the MRI scans.

The following sequences were acquired:

- RS-fMRI BOLD single-shot echo-planar imaging (TR = 3,000 ms, TE = 30 ms, flip angle = 89°, 64 × 64 matrix, 50 contiguous axial slices 3 mm thick, 140 volumes, acquisition time = 7 min 11 s);
- High-resolution 3D, T1-weighted (T1-3D) MPRAGE sequence (TR = 1,900 ms, TE = 2.93 ms, TI = 900 ms, flip angle = 9°, FOV = 260 mm, matrix = 256 × 256, 176 sagittal slices 1 mm thick, no gap), acquired two times consecutively;
- Dual turbo spin-echo, proton density (PD) and T2-weighted images (TR = 3,320 ms, TE1 = 10 ms, TE2 = 103 ms, FOV = 220 mm, matrix = 384 × 384, 25 axial slices 4 mm thick, 30% gap);
- T1-weighted spin echo sequence acquisition, after administration of gadolinium-based contrast agent (Gd), only in patients (TR = 550 ms, TE = 9.8 ms, FOV = 240 mm, matrix = 320 × 320, 25 axial slices 4 mm thick, 30% gap);

The RS-fMRI scan was acquired before (run1) and after (run2) the execution of a repetitive 25-min motor-practice task. We defined two parameters for functional connectivity: FCb, expressing the session's baseline FC in run1, and FCi, that is motor-task induced or a conditioned FC difference, expressed by the contrast “run2-run1.” The structural sequences were acquired during the task, between run1 and run2.

The task consisted in 30-s blocks of movement, alternating with 30-s blocks of rest for fatigue recovery, as previously described (12). An MRI-compatible stimulus-presentation system, employing goggles, and headphones (VisuaStim Digital system by Resonance Technology Inc.) was used to guide the task with visual cues, such as a color fixing cross flashing between orange (flexion) and white (relax-return to resting position) every 0.5 s to obtain the rate of 1 Hz, preceded by a 3-s tag of either a “Flexion” or “Rest” condition at the beginning of each block. Subjects were previously instructed, in a brief out-of-the-scanner training, on how to perform the task and familiarized with the in-scanner visual cue.

### MRI Analysis

#### Lesion Load

On the PD images we calculated the lesion volume (LV) using a semi-automated technique with the Jim 5.0 software (Xinapse System, Leicester, UK; http://www.xinapse.com). Volumes obtained by a trained operator (VG) were reported in cubic millimeters. We cross-controlled lesions on T2-weighted images to increase the confidence level in lesion identification and individual LV masks were obtained.

#### Brain Volume

T1-3D brain images were processed by means of SIENA/SIENAX software, a fully automated method for measuring cross-sectional brain volumes, freely available as part of FSL (Version 5.0; FMRIB Software Library, http://www.fmrib.ox.ac.uk/fsl/). Each T1-3D volume was reoriented to the standard brain template (MNI-152) of FSL and the co-registered LV mask was used to apply lesion filling (lesion_filling, part of FSL). The SIENAX provided measures of global brain volume (GBV), gray matter volume (GMV) and white matter volume (WMV) at baseline (Tst). SIENA provided the percentage estimated brain volume differences between Tst and T6m.

#### Functional Connectivity

**Figure 2:**
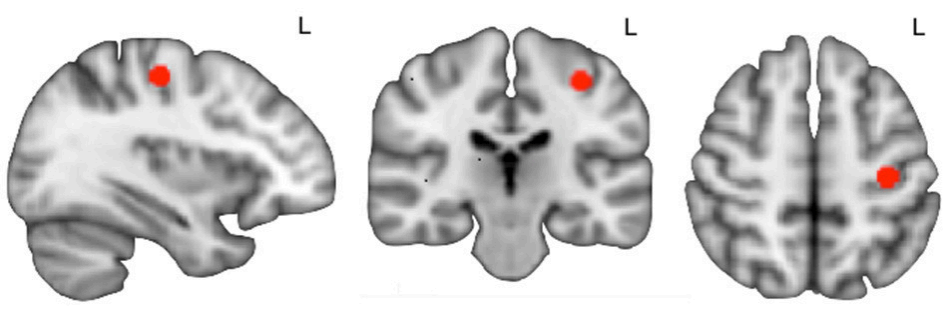
Seed region of interest (in red) overlaid on the T1 standard MNI template.

We performed a seed-to-whole-brain interregional regression analysis using a seed region at the level of the right hand's cortical representation (Figure 2). For the hand's primary motor area identification, we applied a method described by Chris Rorden (20).

In pre-statistics processing we prepared each subject's functional images by motion correction, non-brain substance removal, spatial smoothing, and high-pass filtering. At this stage we also carried out both linear and non-linear registration to obtain functional-to-standard transformation matrices for applying the seed-to-subject's space. For each individual, we obtained at each of the three sessions the following contrast maps: (i) baseline FC (FCb), i.e., run1; (ii) motor task-induced functional connectivity difference (FCi) by the 25-min motor task, i.e., run2-run1. We also obtained ΔFC contrast maps, i.e., differences between FTst and FT6m, for both FCb and FCi.

At a higher, group level, within- and between-session analyses were performed. FC changes between T-3w and Tst represent the no-treatment, test-retest effect; FC changes between Tst and T6m represent treatment-period effect. A within-session group analysis was conducted with one-sample *t*-tests (cluster level *p* < 0.05 corrected for familywise error—FWE) to obtain average group FC maps, at both run1 and run2. In the between-session group analysis, FC maps were entered into two-sample paired *t*-tests (cluster level *p* < 0.05, FWE-corrected).

The anatomical localization of significant clusters was established by the Harvard-Oxford Structural Atlas, the Juelich Histological Atlas, and the Oxford Thalamic Connectivity Probability Atlas included in FSL (http://www.fmrib.ox.ac.uk/fsl/data/atlas-descriptions.html) and visual inspection.

To assess possible relationships between FC changes and clinical improvement, regression/correlation analysis was disposed with only those clinical and behavioral parameters that changed over time. To that end, we used ΔFC maps for both FCb and FCi (ΔFCb and ΔFCi, respectively) and inserted significantly changing parameters as variables of interest in a general linear model (GLM). We also analyzed the relationship between ΔFC maps and tissue damage (LV and gray matter volumes) restricted within the masks obtained by the two-sample paired *t*-tests. All regression/correlation tests were non-parametric, with permutation testing (10,000 permutations) and threshold-free cluster enhancement. Results were considered as significant at cluster level *p* < 0.05, FWE-corrected.

### Statistical Analysis

Descriptive statistics for demographic, clinical and behavioral parameters report the mean and standard deviation, except for EDSS, for which we report the median and range. The comparisons between timepoints for every behavioral parameter were carried out with paired *t*-tests with *p* < 0.05 for null hypothesis rejection.

## Results

### Clinical and Conventional MRI Data

Thirty-six patients were included in the study. Four patients did not complete the follow-up: two of them dropped the study at T-3w, non-tolerant of the session's scanning duration; another two dropped at initiation stage and at 3 m, respectively, due to their unavailability at the protocol time schedule. Thus, data on 32 patients (mean ± SD age = 36.6 ± 7.5 years; 25 females) were available for analysis. Mean disease duration was 8.4 years ± 6.0 SD and the median EDSS score at initiation was 2.0 [range 1.0–6.0]. The other clinical scores are summarized in Table 1.

**Table 1:** Demographic and clinical characteristics of patients at fngolimod therapy initiation (32 patients, where values are reported as the mean ± standard deviation or median [min–max]; *n*, number; *y*, years; *d*, days).

Age, *y*	36.6 ± 7.5
Female/male, *n*	25/7
Disease duration, *y*	8.4 ± 6.0
EDSS score [median (range)]	2.0 [1.0–6.0]
Relapses in previous year, *n* (%)	15 (47)
Treatment start time since previous year relapse, *d* [range]	127 [43–265]
Treatment naïve, *n* (%)	5 (16)

As for the radiological characteristics (Table 2), the baseline mean brain volume was 1,520 cm^3^ ± 49 SD and gray matter volume was 772 cm^3^ ± 35 SD. Two out of the 32 patients denied Gd infusion at all sessions. At FTst, mean T2-LV was 7,011 mm^3^ ± 7.071 SD; Gd-enhancing lesions (Gd+) were observed in 15 out of 30 patients (47%). SIENA showed a median percent brain volume change of −0.27 [−1.49–2.01] between Tst and T6m (one outlier excluded, where brain extraction/segmentation visibly failed).

**Table 2:** Scores obtained in the clinical/neuropsychological assessment and radiological features at initiation (Tst) and after 6 months (T6m) of fingolimod treatment and statistical comparison results (paired *t*-test with threshold of *p* < 0.05); values are reported as the mean ± standard deviation or median [min–max]; ns, not statistically significant).

	**Tst**	**T6m**	***P***
**CLINICAL/NEUROPSYCHOLOGICAL SCORES**			
9-HPT dominant hand, s	19.4 ± 3.8	19.5 ± 3.9	ns
9-HPT non-dominant hand, s	21.6 ± 5.8	21.1 ± 5.3	ns
PASAT 3, s	45 ± 11	50 ± 9	0.016
PASAT 2, s	35 ± 12	41 ± 13	0.011
25-feet walk, s	5.8 ± 1.2	6.0 ± 1.7	ns
MSQoL, score	165 ± 11	165 ± 11	ns
**RADIOLOGICAL FEATURES**			
Brain volume (cm^3^)	1,520 ± 49	−	-
Gray matter volume (cm^3^)	772 ± 35	−	-
T2-lesion volume (cm^3^)	7.011 ± 7.071	−	-
Gadolinium positive lesions, *n* (%)	15 out of 30 (47)	3 out of 30 (10)	-
New T2-lesions, *n* (%)	-	14 out of 32 (44)	-
Percentage brain volume change (%)	-	−0.27 [−1.49–2.01]	-

*9-HPT, 9-hole peg test; PASAT, Paced Auditory Serial Addition Test; MSQoL, MS Quality of Life*.

After 6 months of therapy, only PASAT showed a significant improvement (Table 2). At T6m, 14 out of 32 patients (44%) showed new T2 lesions with respect to the Tst and 3 of them (10%) showed Gd+ lesions (Table 2).

### Functional Connectivity Changes

**Figure 3:**
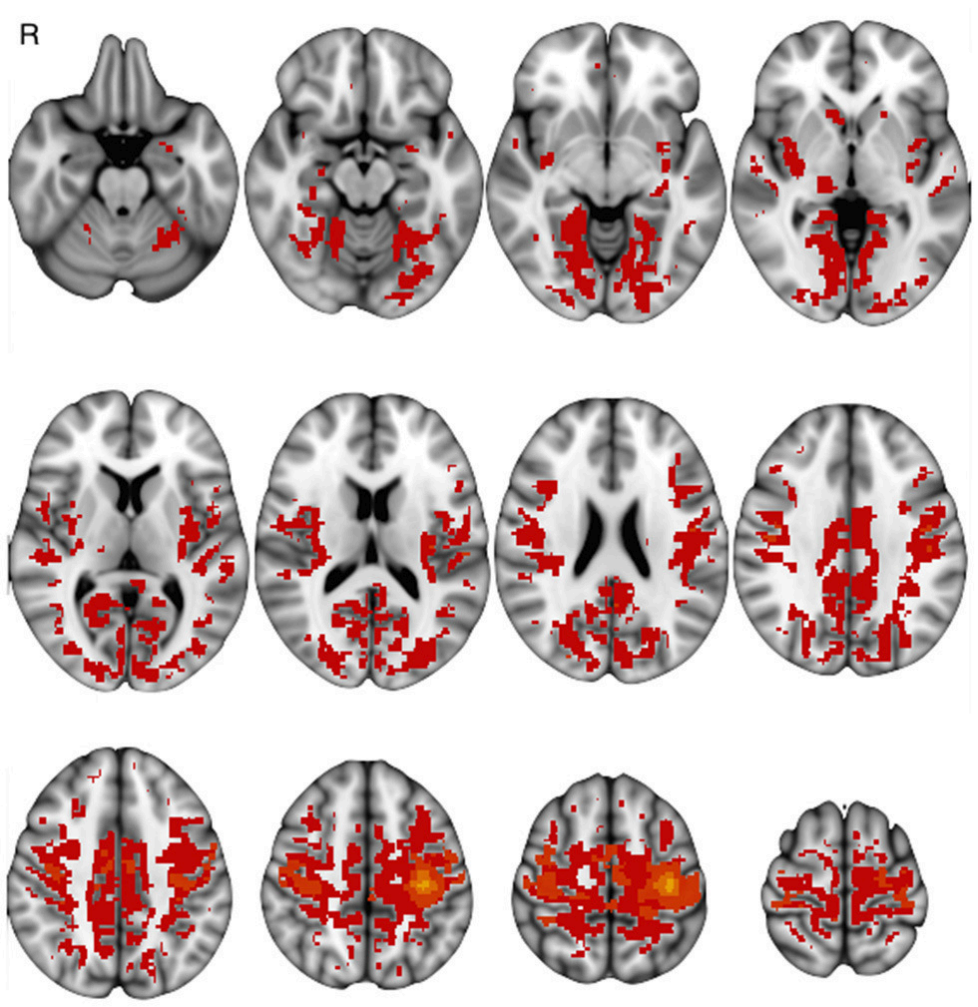
One-group baseline functional connectivity (*p* < 0.05 at cluster level) at Tst session (*p* < 0.05 at cluster level).

Basal FC showed a similar pattern across sessions: FCb involved the sensorimotor cortex and the supplementary motor area and further areas in the temporal, parietal and occipital cortices as well as in the basal ganglia and cerebellum, bilaterally (Figure 3).

**Figure 4:**
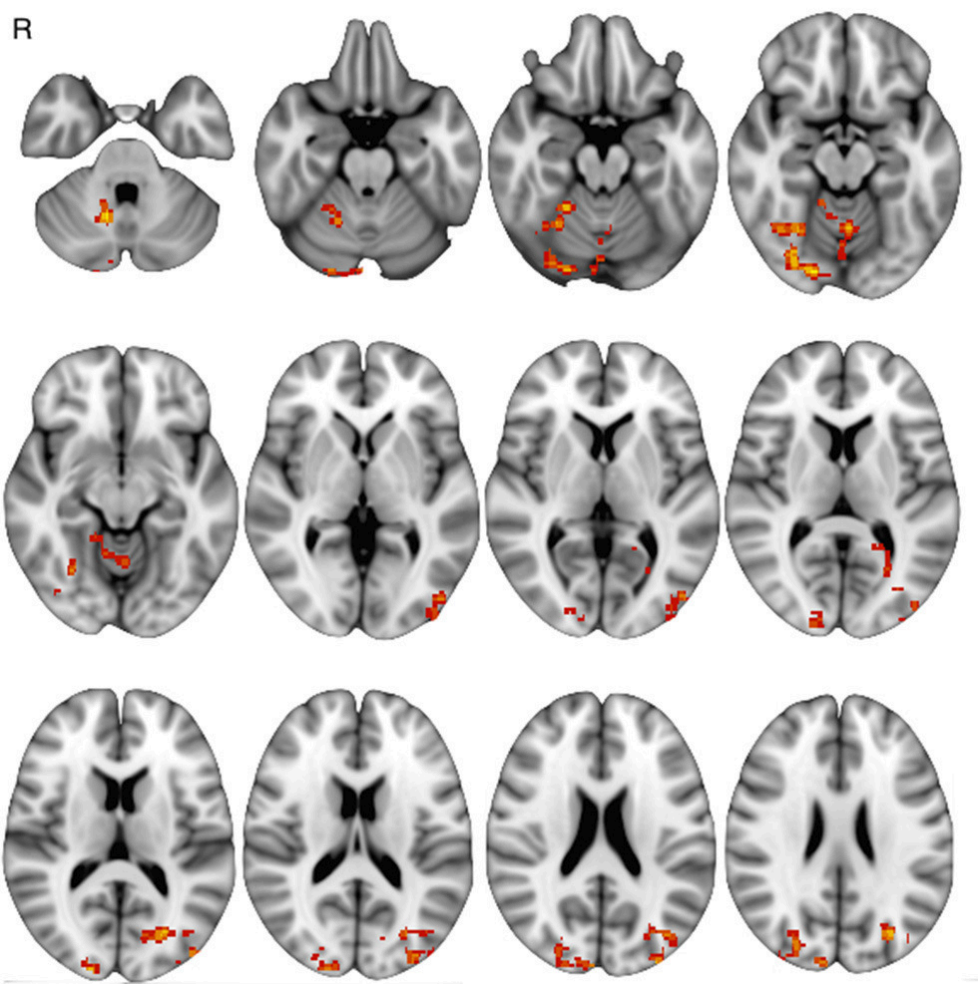
Baseline functional connectivity decrease at T6m, with respect to Tst session (*p* < 0.05 at cluster level).

The comparison of FCb between the first two sessions ΔFCb, i.e., the T-3w and Tst, did not show any statistically significant change, indicating a null test-retest effect. On the other hand, the comparison between Tst and T6m sessions, which represents therapy-associated modifications, showed a significant FCb reduction in the cortical occipito/parietal areas bilaterally, as well as in the vermis and right cerebellar hemisphere (Figure 4).

**Figure 5:**
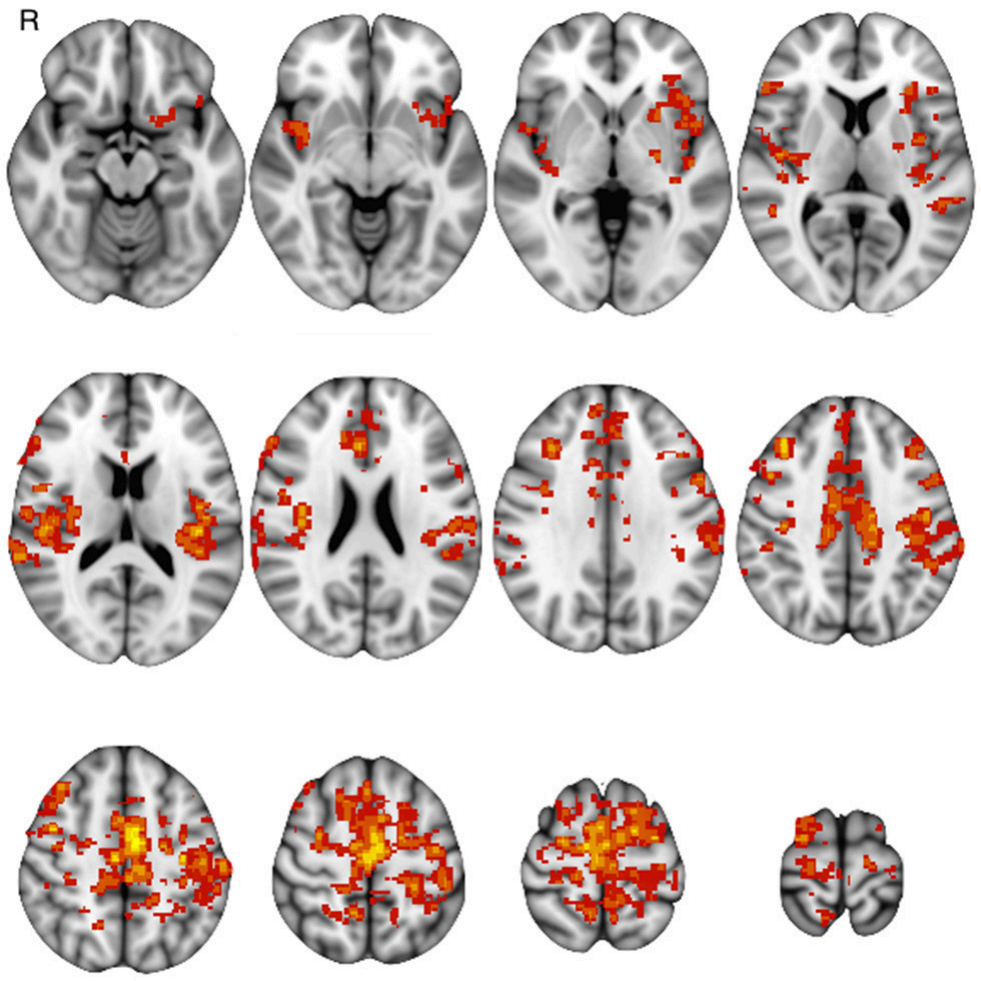
One-group results of post training functional connectivity increase at T6m (*p* < 0.05 at cluster level).

**Figure 6:**
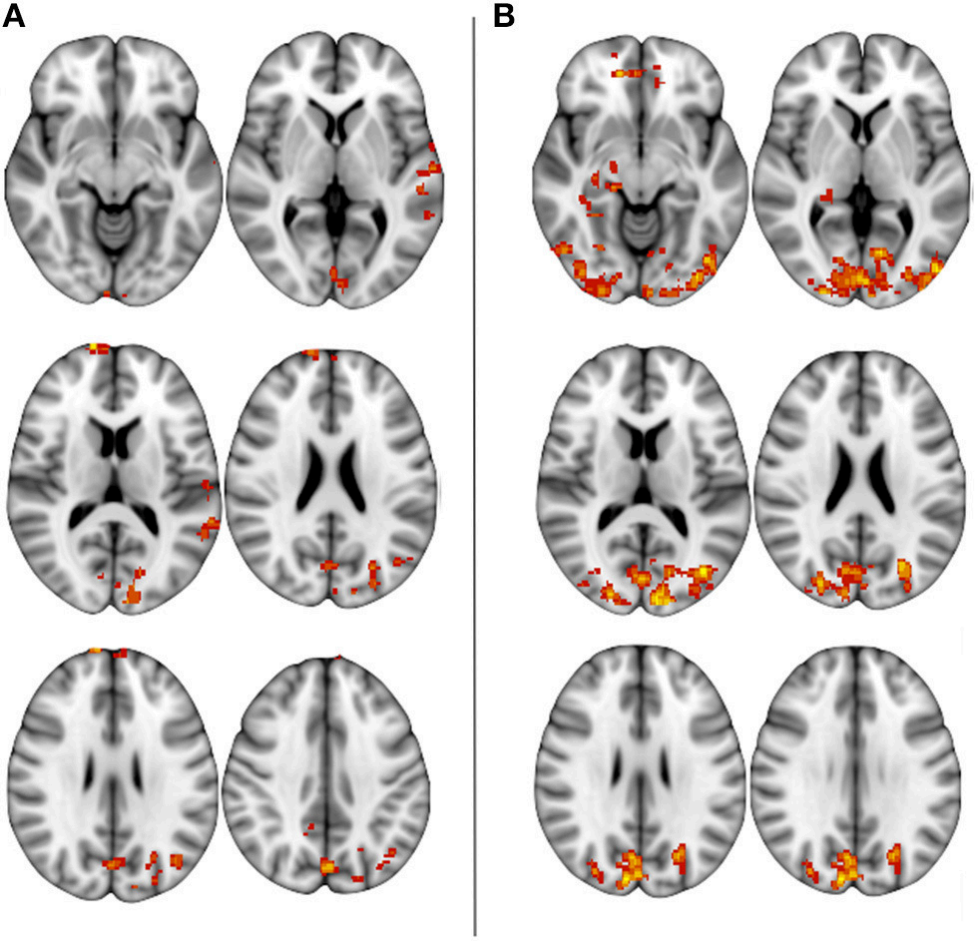
Between-session, post-training differences in FC increase (*p* < 0.05 at cluster level): **(A)** greater FCi at T-3w than Tst, **(B)** greater FCi after 6-months of treatment then at Tst.

Motor-task induced FC changes (FCi) showed a statistically significant main effect in all examined sessions, involving not only areas of the sensorimotor network, but also posterior cortical areas (cuneus and precuneus) and areas of the prefrontal and temporal cortices; the main group-effect is shown in Figure 5. No main effect was significant for the inverse contrast (run1 > run2). The between-session comparison showed significant FCi differences (ΔFCi): at T-3w difference was greater than at Tst in cuneus and precuneus bilaterally, in left temporal and in prefrontal cortices; at T6m difference was also greater than at Tst, still in cuneus and precuneus but more extended, including the mesial frontal cortex and areas in the right hippocampal and parahippocampal gyri (Figure 6).

### Correlations Between FC Changes and Clinical/Radiological Features

PASAT3 and PASAT2 were the only clinical parameters that changed significantly, showing an improvement over time as documented by increased scores from the initiation session to 6 months; we inserted percentage changes in both tests as covariates of interest in the GLM.

**Figure 7:**
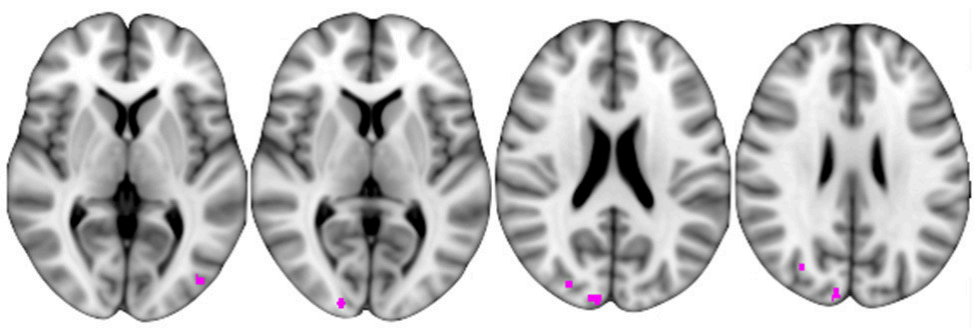
Correlation between baseline functional connectivity decrease at T6m with respect to Tst session and PASAT3 increase at the same period.

The ΔFCb, i.e., FCb differences between Tst and T6m, showed a significant inverse relation with the PASAT3 percentage change, in the posterior cortical parietal and occipital areas (Figure 7); this indicates that the greater the FCb reduction in those areas, the better the improvement in the performance. No significant relationship was found between ΔFCb and PASAT2 percentage changes.

No significant relationship was found between ΔFCi and PASAT percentage changes. No significant relationship was found between ΔFCb or ΔFCi and the structural damage (T2 LV and gray matter volume).

Furthermore, to explore whether the observed FC changes were related to conventional MRI measures of disease activity, we divided patients into subgroups according to the following criteria (1) presence of Gd+ lesions at initiation; (2) new T2 and/or presence of Gd+ lesions at follow-up observation. No significant differences in either ΔFCb or ΔFCi were observed between the Gd+ (*N* = 15) and Gd negative (*N* = 15) cases at initiation, as well as between patients developing new T2 lesions during the study (*N* = 14) and those remaining stable (*N* = 18) in unpaired two-sample comparisons.

## Discussion

This study shows significant changes in functional connectivity after 6 months of fingolimod therapy.

Our study cohort's clinical and anamnestic characteristics at recruitment are in line with those reported in post-marketing studies (21, 22) and those required for the prescription of Fingolimod (14).

We found that baseline FC was higher at the initiation stage and diminished after 6 months of treatment in posterior (parieto/occipital) cortex and cerebellum. In addition, the inter-session FCi, after 25 min of motor conditioning, increased in posterior cortical areas (cuneus and precuneus), in the medial frontal cortex and in the right hippocampus/parahippocampus.

Changes in baseline FC after 6 months of treatment with fingolimod were evident in specific areas. The posterior cortical areas belong, in part, to the default-mode network (DMN) (4), whose modulation has been related with improvement in cognitive tasks after training in MS (23, 24). Additionally, their activity has been demonstrated to inversely correlate with task-based cortical and subcortical motor areas (25). Areas of the posterior median cortex, i.e., the cuneus and precuneus, are also acting as network hubs whose connectivity is modulated in relation to several brain networks (26).

Therefore, changes after fingolimod therapy might be the result of FC modifications in posterior medial cortical areas and their reciprocal modulation with the sensorimotor network.

The increased FCi likely represents a higher reactivity in modulating FC, as response to the motor conditioning (12). This kind of response occurred in cortical areas whose spontaneous, resting fluctuations are known to relate to the corresponding motor cortex spontaneous activity: the right hippocampus and parahippocampal areas are generally related to long-lasting memory processes and learning through long-term potentiation (27), while the pre-frontal areas are part of the executive-function network (28). Thus, the FCi observed in these areas after the motor task could express the consolidation of newly developed patterns of motor execution.

Taken together, FCb and FCi modulation could be interpreted as an expression of plasticity at the level of large-scale RS networks, promoted or influenced by the fingolimod therapy.

We also found a correlation between the decrease in basal FC and an increase in the PASAT scores after fingolimod. This phenomenon may be interpreted as an improvement of cognitive function related to the effect of the drug, as suggested. In fact, a recent multi-center prospective study (29), including MS patients treated with IFN beta 1b and Fingolimod, demonstrated that both treatments were associated with significant improvement in the cognitive parameters: fingolimod showed the best effect on PASAT, whereas IFN beta 1b showed the best effect on the Symbol Digit Modalities Test (SDMT).

Due to the study design, we cannot exclude that this effect is, at least in part, due to a learning effect. Nevertheless, a recent study suggests that the short-term practice effects on PASAT are related to brain volume, disease severity, and age, and have clinically meaningful prognostic implications (30). Therefore, we can claim that the increase of the PASAT score seen at month 6, implying a cognitive improvement and/or the retention of the practice effect, can be interpreted as a positive phenomenon. Recently, Rocca et al. reported FC abnormalities in MS and their correlations with disability and cognitive impairment (31): they showed that reduced FC correlated with better neuropsychological test battery performance in a large cohort of MS subjects. The reduction in FC after fingolimod in our study might be the expression of a beneficial effect of the drug in allowing adaptive neuroplastic changes and preventing functional overload and network collapse (32, 33). The finding that changes in PASAT scores were related to changes in functional measures of spontaneous brain activity at rest would suggest that, at least in part, neuropsychological improvement may not exclusively reflect a learning effect.

A longitudinal correlational study (34) suggested that PASAT is particularly sensitive to inflammatory activity, indicated by contrast agent enhancement, in otherwise clinically stable MS patients. Interestingly, the FC changes that we observed do not seem to exclusively depend on disease activity decrease, as seen on conventional MRI (i.e., no significant differences in patients with persistent activity vs. those without MRI activity), but they may also be due to fingolimod's immuno-modulatory effects on inflammation mediated by the innate-immunity. Fingolimod's proven anti-inflammatory effect is related to its action as a functional antagonist of the sphingosine-1-phosphate receptor 1, determining its internalization; in doing so, it impedes lymphocyte migration into the central nervous system (35–37).

Evidence from experimental studies suggest that the immune system plays a central role in modulating learning, memory and neural plasticity. The overexpression of immune diffusible mediators (38, 39) may contribute to the disruption of synaptic plastic processes, thus impairing the ability to encode and retain information (38, 40, 41). Damage of the connections between brain regions, occurring in MS inflammation, may hinder the spatial and temporal coherence of signal transmission (38, 42), necessary for plasticity to effectively drive recovery (43).

In sum, the described different combined effects of fingolimod suggest that, by toggling negative immune and inflammatory factors, it may promote the restoration of synaptic plasticity and the facilitation of recovery.

### Limits

A limit in this study is the lack of a control group of healthy subjects or a group of untreated patients. A healthy subject group would have permitted to characterize as patient-specific the observed FC changes. However, evidence from previous cross-sectional controlled studies show that MS patients and healthy subjects have different baseline connectivity in most resting-state networks (7) and different conditioned reactivity for movement-related networks (12). On the other side, assigning a group of untreated MS patients is unethical because it is equivalent to a denial of treatment against the patient's best interest. This is a study design issue that we addressed by the longitudinal model.

A second limit of the study is that the anti-inflammatory effects of fingolimod were evaluated only by means of the conventional MRI. The application of other techniques (e.g., positron emission tomography) might have evaluated the anti-inflammatory effects of fingolimod even on the inflammation of brain tissue outside the lesion areas.

A third limit is the assessment of the cognitive status only by the PASAT score, which cannot be considered a global measure of cognitive function. PASAT score, however, has proven to be a sensitive measure for detecting cognitive impairment in MS (44). Comparatively, our results are in line with studies showing a positive effect of disease-modifying drugs, fingolimod in particular, on cognitive function, after 18 months of treatment (29). Furthermore, PASAT was reported as a difficult and demanding cognitive test, relatively more difficult than the SDMT, and sensitive even to early cognitive abnormalities in patients where room for improvement is still limited (45, 46).

## Conclusion

The study shows that the anti-inflammatory effect of 6 months of therapy with fingolimod determines a brain functional rearrangement that has been observed through two different modalities, i.e., basal and task-conditioned functional connectivity. Both have evidenced the prevalent involvement of the posterior cerebral cortex. Moreover, the significant association between functional connectivity changes in those brain areas and cognitive improvement points to a beneficial effect of fingolimod-associated neuroplastic changes.

Taken together, these results suggest that neuroplastic changes after fingolimod therapy mainly consist in a significant effect in cortical areas located outside the sensorimotor system, but which are functionally integrated or related with it. The RS-fMRI changes we observed after therapeutic intervention may be considered as an expression of neuroplastic changes associated with the anti-inflammation effect, at least at the organizational level of the brain's resting-state functional connectivity.

## Data Availability

The datasets generated for this study are available on request to the corresponding author.

## Ethics Statement

This study was carried out in accordance with the recommendations of the Ethical Code, Ethical Committee of the Azienda Policlinico Umberto I - Sapienza University of Rome, Viale del Policlinico 155, 00161 (RM), Rome, Italy, with written informed consent from all subjects. All subjects gave written informed consent in accordance with the Declaration of Helsinki. The protocol was approved by the abovementioned Ethical Committee.

## Author Contributions

NP contributed to study design, experimental setting, subject recruitment, MRI acquisition, method definition, data analysis, statistics, and manuscript editing. LD and CM contributed in data interpretation and manuscript editing. FT and VG-Q did data quality evaluation and lesion assessment. MC did MRI acquisition. MG and FD did patient recruitment and clinical and neuropsychological assessment. CP did the study design and recruitment and, together with PP, study supervising and manuscript editing.

### Conflict of Interest Statement

NP received speaker fees from Biogen and mission support from Genzyme and Novartis. CP received consulting and lecture fees from Sanofi-Aventis, Biogen Idec, Bayer Schering, Merck Serono, and Novartis. He also received research funding from Novartis, Sanofi-Aventis, Merck Serono, and Bayer Schering. The remaining authors declare that the research was conducted in the absence of any commercial or financial relationships that could be construed as a potential conflict of interest.
